# Understanding COVID‐19 Vaccine Acceptance Among Iranian Pregnant Women: Insights From a Multicenter Cross‐Sectional Study

**DOI:** 10.1002/hsr2.70176

**Published:** 2024-11-11

**Authors:** Azita Fathnezhad‐Kazemi, Lila Hadipour, Raziyeh Pirami, Somayyeh Khazaeian

**Affiliations:** ^1^ Department of Midwifery, Women's Reproductive and Mental Health Research Center Tabriz Medical Sciences Tabriz Iran; ^2^ Vice‐Chancellor of Health Affairs Khalkhal University of Medical Sciences Khalkhal Iran; ^3^ Vali‐e‐Asr Hospital Birjand University of Medical Sciences Birjand Iran; ^4^ Pregnancy Health Research Center, Department of Midwifery Zahedan University of Medical Sciences Zahedan Iran

**Keywords:** acceptance, COVID‐19, Health Belief Model, pregnant women, vaccination

## Abstract

**Background and Aims:**

Despite significant advancements in COVID‐19 vaccine research and development, hesitancy in its acceptance, particularly among pregnant women, is recognized as a health threat. Based on the Health Belief Model, this study aimed to identify the factors influencing vaccine nonacceptance among pregnant women in the cities of Zahedan and Tabriz, Iran.

**Methods:**

The present research was a multicenter cross‐sectional study conducted among pregnant women in two Iranian provinces, Zahedan and Tabriz, covering both high and low fertility regions. The study was conducted from February 2022 to August 2022. The sample size comprised 650 pregnant women attending selected healthcare centers. Data collection tools included questionnaires on sociodemographic characteristics, awareness, vaccine acceptance, and health beliefs related to vaccination. Data were analyzed using descriptive and analytical methods, including Analysis of variance (ANOVA), Independent *t*‐test, and multivariable logistic regression.

**Results:**

Among the 650 pregnant women, the vaccine acceptance rate was 47.4% and 52.6% of pregnant women reported vaccine nonacceptance during pregnancy. The logistic regression model indicated that the odds of vaccine acceptance increase with maternal employment (OR = 11.53, 95% CI: 4.81–27.63), higher maternal education levels (OR = 6.97, 95% CI: 3.11–15.63), higher income (OR = 1.60, 95% CI: 1.07–2.55), and increased awareness levels (OR = 1.37, 95% CI: 1.03–1.52). Additionally, the odds of vaccine acceptance increase with increased perceived susceptibility (OR = 2.55, 95% CI: 0.47–3.76), increased perceived benefits (OR = 2.22, 95% CI: 1.06–4.61), increased perceived barriers (OR = 0.42, 95% CI: 0.18–1.12), and increased cues to action (OR = 2.01, 95% CI: 1.03–3.92).

**Conclusion:**

Our study underscores the multifaceted nature of COVID‐19 vaccine acceptance among pregnant women, with socioeconomic, awareness‐related, and psychological factors all playing pivotal roles. These findings can inform targeted interventions and strategies aimed at increasing vaccine acceptance in this vulnerable population, ultimately contributing to enhanced maternal and fetal health during the ongoing pandemic.

## 1 Background

The COVID‐19 vaccination is considered one of the greatest achievements in 21st‐century medical science, rapidly deployed worldwide. It is hailed as the primary tool for combating the spread of the COVID‐19 virus and reducing the prevalence of the disease [1, 2, 3]. The successful implementation of the COVID‐19 vaccination program is not solely dependent on the effectiveness and efficiency of the vaccine. In addition to having a robust and integrated healthcare system, it requires strong public trust and societal acceptance of the vaccine [1]. In worldwide vaccination initiatives, achieving widespread acceptance and comprehensive coverage is pivotal for the success of vaccination programs [4]. However, concerns and doubts about vaccination, particularly among pregnant women, are common, partly due to the unique circumstances of pregnancy [5].

Despite limited data, most medical organizations recommend COVID‐19 vaccination during pregnancy due to the potential risks of contracting COVID‐19 for both the mother and the fetus [6, 7]. In addition to the adverse pregnancy‐related outcomes associated with COVID‐19, another critical aspect is how this disease, like other health issues, is influenced by ignorance, poverty, and a lack of healthcare infrastructure [8]. Therefore, COVID‐19 has the potential to increase maternal mortality in less‐developed countries because, despite significant advancements in vaccine research and development, vaccine hesitancy is recognized as a threat to public health [9].

The results of a systematic review encompassing data on COVID‐19 vaccine acceptance in 33 countries worldwide indicate a vaccination acceptance rate of less than 70%, with notably lower acceptance rates, particularly in the Middle East [10]. Furthermore, Iranian researchers have reported a moderate level of willingness to receive the COVID‐19 vaccine [11]. According to the researchers' report, not only do attitudes toward vaccine use and COVID‐19 vaccine acceptance vary among women globally, but these attitudes are also influenced by various factors [12].

Prior research on most available vaccines in society has shown that a wide range of factors, including the compulsory nature of vaccines, their random temporal associations with adverse health outcomes, unfamiliarity with vaccine‐preventable diseases, and mistrust of vaccine effects, contribute to vaccine hesitancy and doubt regarding vaccine use [13].

Regarding the coronavirus disease, in addition to the reasons mentioned above, the novelty of the disease and its wide spectrum of symptoms have created challenges and complexities in vaccine acceptance within communities. The emergency use authorization of these vaccines and concerns about the long‐term benefits of the vaccines, which remain uncertain, have also added to the complexity [14].

On the other hand, vaccination in pregnant women has always been associated with multiple and increased challenges. For example, awareness among pregnant mothers about common diseases such as influenza has been limited, and despite recommendations for vaccination, the vaccination rate has been low due to fears of harming the fetus [15, 16, 17].

Limited data and evolving recommendations on COVID‐19 vaccination during pregnancy, coupled with widespread, sometimes inaccurate information on social media, have made many pregnant women significantly concerned about the unknown long‐term effects of COVID‐19 vaccines during pregnancy, leading to vaccine hesitancy [18].

However, disease control programs through vaccination are only successful when the vaccination acceptance and coverage rates within the community are high [10]. While vaccination is effective, there are still challenges with achieving sufficient and comprehensive vaccination coverage for achieving immunity or prevention. Reluctance to accept vaccines can seriously impact global efforts to control the COVID‐19 virus [11].

Considering that vaccine acceptance is regarded as a form of health behavior for disease prevention, the use of educational models such as the Health Belief Model can serve as an appropriate framework for understanding and promoting health‐related behaviors. This model can explain acceptance or refusal behaviors related to vaccination based on various positive and negative factors that individuals consider [19].

Due to the novelty of the COVID‐19 disease, the unpredictable nature of vaccine acceptance in different societies, and the absence of prior studies utilizing the Health Belief Model to identify factors influencing COVID‐19 vaccine hesitancy among pregnant women in Iran, this study was conducted. The objective of this study was to assess the influential factors contributing to vaccine hesitancy based on the Health Belief Model in the cities of Zahedan and Tabriz, which represent contrasting poles in terms of high and low birth rates in Iran [20].

## 2 Methods

### 2.1 Study Design and Sampling

The present study is a multicenter cross‐sectional study conducted from February 2022 to August 2022 in two cities, Tabriz and Zahedan, Iran. Tabriz is the capital of East Azerbaijan Province and is located in northwestern Iran, while Zahedan is the capital of Sistan and Baluchestan, and is situated in southeastern Iran. The study population included all pregnant women attending healthcare centers in both cities. Inclusion criteria were: literacy in reading and writing, pregnancy of 12 weeks or more, Iranian nationality, absence of any physical, mental, or psychological illnesses based on medical records, no history of COVID‐19 infection, no completion of vaccination before pregnancy, and a willingness to continue participation in the study and complete the questionnaires, with noncompliance being one of the withdrawal criteria.

The sample size for this study was calculated based on the information obtained from the study by Ayhan et al. [21] in Turk, considering a 37% vaccine acceptance rate among pregnant women, a power of 90%, a confidence interval of 95%, and an error rate of 15% around the prevalence, with the following formula. Using these values, the initial sample size was calculated to be 296 individuals. An additional 10% was added to the sample size to increase the study's accuracy, resulting in a final sample size of 325 individuals for each city.

$$N=\frac{{\left(Z_{1}-\frac{\alpha }{2}\right)}^{2}\times p(1p)}{d^{2}}.$$

After obtaining ethical approval and authorization from university presidents and healthcare authorities in the cities of Zahedan and Tabriz, a double‐stage sampling was carried out. We chose 21 healthcare centers in Tabriz (comprising 87 healthcare centers) and 8 healthcare centers in Zahedan (comprising 32 healthcare centers, using randomizer software (www.random.org), after that, the eligible participants were selected with Convenience Sampling method and they entered the study if they wanted and based on inclusion criteria.

### 2.2 Data Collection Tools

The data collection tools in this section of the study consist of the following components.

#### 2.2.1 Demographic and Background Information

This includes 26 questions covering demographic variables such as age, education, occupation, age of pregnancy, and number of pregnancies.

#### 2.2.2 Awareness Questionnaire

An 8‐item questionnaire related to awareness was adapted from the Saifol‐Islam and Mannan studies [22, 23]. Questions were scored as 2 for “yes,” 1 for “somewhat,” and 0 for “no.” The score range for this questionnaire is between 0 and 16, with higher scores indicating higher awareness.

#### 2.2.3 Vaccine Acceptance Questionnaire

An 18‐item questionnaire about vaccine acceptance was designed in such a way that a “yes” response received a score of 1, a “no” response received a score of 0, and the last question awarded a score of 2 if the individual had received the vaccine. This questionnaire was developed based on the Ayhan [21] study. The score range for this questionnaire is between 0 and 19, with higher scores indicating greater vaccine acceptance.

#### 2.2.4 Health Belief Structure Questionnaire

This questionnaire was developed based on the Tao et al. [24] study and consists of 14 items related to health belief constructs, including 2 items on perceived susceptibility, 2 items on perceived severity, 3 items on perceived barriers, 3 items on perceived benefits, and 4 items on cues to action. Items in the health belief questionnaire were divided into a binary scale of “agree” (1) and “disagree” (0) based on a 2‐point Likert scale. The score range for perceived susceptibility and severity is 0–2, perceived barriers and benefits are 0–3, and cues to action are 0–4. Higher scores in each component indicate a higher level of understanding in that component.

To assess the reliability and validity of the questionnaires, both qualitative and quantitative methods were employed. In the qualitative phase, the questionnaires were reviewed by five faculty members from Zahedan and Tabriz Medical Universities, and necessary adjustments were made. In the quantitative phase, the content validity ratio and content validity index were used to determine content validity. For reliability assessment, a test–retest method was employed, where the questionnaires were administered to 20 participants who were not part of the main sample, with a 2‐week interval between administrations.

This comprehensive data collection approach ensures that the study gathers accurate and reliable information on various aspects of awareness, vaccine acceptance, and health beliefs among pregnant women.

### 2.3 Data Analysis

Data analysis was performed in SPSS version 24.0 (IBM Corp., Armonk, NY, USA). Descriptive statistics (i.e., frequencies, percentages, mean, SD) were used to present the data. Differences in basic variables between two groups and more than two groups were evaluated using an independent *t*‐test, analysis of variance, and chi‐square test, respectively. Predictors of vaccine acceptance were evaluated based on multivariable logistic regression. The normality of the data was also determined by the Shapiro–Wilk test. All *p* values were two‐sided. A significance level of *p* < 0.05 is considered.

### 2.4 Ethical Considerations

This study was approved by the Ethics Committee of Zahedan University of Medical Sciences (code number: IR.ZAUMS.REC.1400.358). The study was conducted by the Helsinki Declaration of 1975. After explaining the study objectives, informed consent was obtained from all participants.

## 3 Results

In this study, a total of 650 pregnant women from the cities of Zahedan and Tabriz participated. The results showed that the mean (standard deviation) age of the participants was 26.85 (6.02) years. Among the women from Zahedan, 53% had three or more pregnancies, while in Tabriz, this percentage was 42% had only one pregnancy. Significant correlations were found between the age of women, educational level, employment status, age of pregnancy, vaccine acceptance, and awareness scores (*p* < 0.05) (Table 1).

**Table 1:** Acceptance of a COVID‐19 vaccine of pregnant women by sociodemographic characteristics in two cities.

Variable	*N* (%)	Zahedan (*n* = 325) *n* (%)	Tabriz (*n* = 325) *n* (%)	Acceptance of a COVID‐19 vaccine		Awareness Mean (SD)
				No, *n* (%)	Yes, *n* (%)	
Mother's age groups (year) Mean (SD) 26.85 (6.02)						
> 20	101 (15.5)	72 (22.1)	29 (8.9)	71 (20.8)	30 (9.7)	7.63 (2.03)
20–29	374 (57.5)	218 (67)	156 (48)	197 (57.6)	177 (57.4)	8.40 (2.14)
30–39	156 (24.0)	34 (10.4)	122 (37.5)	69 (20.2)	87 (28.2)	8.03 (2.06)
≥ 40	19 (2.9)	1 (0.5)	18 (5.6)	5 (1.4)	14 (4.7)	7.52 (2.36)
*p* value		< 0.001^a^		< 0.001^a^		< 0.001^b^
Mother's educational status						
Primary school	97 (14.9)	79 (24.3)	18 (5.5)	80 (23.4)	17 (5.5)	7.71 (1.74)
Secondary school	184 (18.8)	113 (34.7)	71 (21.8)	158 (46.1)	26 (8.4)	7.86 (2.29)
Diploma	251 (46.6)	98 (30.1)	153 (47.0)	93 (27.1)	158 (51.3)	8.43 (2.13)
University	118 (19.7)	35 (10.9)	83 (25.7)	11 (3.4)	107 (34.8)	9.60 (2.12)
*p* value		< 0.001^a^		< 0.001^a^		< 0.001^b^
Mother's employment status						
Housewife	559 (86.0)	297 (91.4)	262 (80.6)	333 (97.4)	226 (73.4)	8.30 (2.14)
Employed	91 (14.0)	28 (8.6)	63 (19.4)	9 (2.6)	82 (26.6)	9.36 (2.3)
*p* value		< 0.001^a^		< 0.001^a^		< 0.001^c^
Spouse's educational status						
Primary school	135 (20.8)	87 (26.8)	48 (14.8)	79 (23.1)	56 (18.2)	8.17 (1.84)
Secondary school	211 (32.5)	133 (40.9)	78 (24.0)	125 (36.5)	86 (27.9)	8.20 (2.15)
Diploma	199 (30.6)	62 (19.1)	137 (42.2)	95 (27.8)	104 (33.8)	8.66 (2.39)
University	105 (16.2)	43 (13.2)	62 (19.1)	43 (12.6)	62 (20.1)	8.88 (2.23)
*p* value		< 0.001^a^		0.004^a^		0.013^b^
Spouse's employment status						
Jobless	33 (5.1)	21 (6.5)	12 (3.7)	24 (3.7)	9 (1.4)	8.03 (1.64)
Employed	527 (81.1)	262 (80.6)	265 (81.5)	278 (42.8)	249 (38.3)	8.47 (2.25)
Self‐employed	90 (13)	42 (12.9)	90 (13.8)	40 (6.2)	50 (7.7)	8.48 (2.07)
*p* value		0.238^a^		0.021^a^		0.529^b^
Number of pregnancies						
1	178 (27.3)	41 (12.6)	137 (42.2)	92 (26.9)	86 (29.9)	8.50 (1.97)
2	211 (32.4)	110 (33.8)	101 (31)	115 (33.6)	96 (31.2)	8.59 (2.27)
≥ 3	261 (40.3)	174 (53.6)	87 (26.8)	135 (39.5)	126 (38.9)	8.28 (2.34)
*p* value		< 0.001^a^		0.0573^a^		0.314^b^
Gestational age (weeks) Mean (SD) 25.44 (7.71)						
≥ 14	27 (4.2)	12 (3.7)	15 (4.6)	14 (4.1)	13 (4.2)	9.37 (2.78)
15–28	398 (61.2)	242 (74.5)	156 (48.0)	227 (66.4)	171 (55.5)	8.55 (2.13)
29≥	225 (34.6)	71 (21.8)	154 (47.4)	101 (29.5)	124 (40.3)	8.32 (2.17)
*p* value		< 0.001^a^		0.014^a^		0.040^b^
Income status						
Less than enough	414 (63.7)	197 (60.6)	217 (66.8)	206 (60.2)	208 (67.5)	7.85 (2.11)
enough	229 (35.2)	128 (39.4)	101 (31.1)	135 (39.5)	94 (30.5)	8.41 (2.00)
More than enough	7 (1.1)	0 (0)	7 (1.1)	1 (0.3)	6 (1.9)	8.48 (2.30)
*p* value		0.004^a^		0.010^a^		0.724^b^
History of COVID‐19 in family members						
Yes	230 (35.4)	117 (36.0)	113 (34.8)	117 (34.2)	113 (36.7)	8.45 (2.27)
No	420 (64.4)	208 (64.0)	212 (65.2)	225 (65.8)	195 (63.3)	8.44 (2.16)
*p* value		0.743^a^		0.509^a^		0.947^c^

^a^ Chi‐squared test.

^b^ Analysis of variance (ANOVA).

^c^ Independent *t*‐test.

63.4% (206 participants) of pregnant women in Zahedan and 40.9% (133 participants) in Tabriz reported that they had not heard anything about the COVID‐19 vaccine in the past. In total, 78% (507 participants) stated that they had insufficient knowledge in this regard. 70.1% of pregnant women in Zahedan expressed unwillingness to receive the vaccine during pregnancy if recommended. In contrast, in Tabriz, this percentage was 35% (Table 2).

**Table 2:** COVID‐19 vaccine acceptance rates comparisons of the answers of the participants in two cities.

Questions	Answer	Total	Zahedan	Tabriz	*p* value^a^
Have you ever been vaccinated?	Yes	592 (91.1)	295 (90.8)	297 (91.4)	< 0.001
	No	28 (8.6)	0 (0.0)	28 (8.6	
Have you been vaccinated in the last 5 years?	Yes	323 (49.7)	165 (50.8)	167 (51.4)	0.583
	No	237 (50.3)	160 (49.2)	158 (48.6)	
Was the influenza vaccine recommended in the present pregnancy?	Yes	502 (77.2)	304 (93.5)	198 (60.9)	< 0.001
	No	148 (22.8)	21 (6.5)	127 (39.1)	
If the influenza vaccine was recommended, would you have been vaccinated in the present pregnancy?	Yes	302 (46.8)	97 (29.8)	207 (63.7)	< 0.001
	No	346 (53.2)	228 (70.2)	118 (36.3)	
Have you been vaccinated for influenza in the present pregnancy?	Yes	130 (20.0)	72 (22.2)	58 (17.5)	0.170
	No	520 (80.0)	253 (77.8)	276 (82.2)	
Was the tetanus vaccine Recommended in the present pregnancy?	Yes	473 (27.2)	229 (70.5)	224 (75.1)	0.186
	No	177 (27.2)	96 (29.5)	81 (24.9)	
Have you been vaccinated for tetanus in the present pregnancy	Yes	618 (95.1)	297 (91.4)	321 (98.8)	< 0.001
	No	32 (4.9)	28 (8.6)	4 (1.12)	
Are you going to have your baby vaccinated after birth?	Yes	612 (94.2)	287 (88.3)	325 (100)	< 0.001
	No	38 (5.8)	38 (11.7)	0 (0)	
Do you have a high risk of COVID‐19 transmission at work?	Yes	44 (6.8)	19 (5.8)	25 (7.7)	0.349
	No	606 (93.2)	306 (94.2)	300 (92.3)	
Did you have close contact with a COVID‐19‐positive person?	Yes	166 (25.5)	66 (20.3)	100 (30.7)	0.0487
	No	484 (74.5)	259 (79.7)	225 (69.3)	
Did you care about hand hygiene during the pandemic?	Yes	539 (82.9)	240 (73.8)	299 (92.0)	< 0.001
	No	111 (17.1)	85 (26.2)	26 (8.0)	
Did you care about social Distancing during the pandemic?	Yes	469 (72.2)	225 (69.2)	244 (75.1)	0.096
	No	181 (27.8)	81 (24.90)	100 (30.8)	
Did you care about using a mask During the pandemic?	Yes	396 (60.9)	81 (24.9)	315 (96.9)	< 0.001
	No	254 (39.1)	244 (75.1)	10 (3.1)	
Did you have COVID‐19 in this pregnancy?	Yes	17 (2.6)	12 (3.7)	5 (1.5)	0.085
	No	633 (97.4)	313 (96.3)	320 (98.5)	
Have you heard about the COVID‐19 vaccine before?	Yes	311 (47.8)	119 (36.6)	192 (59.1)	< 0.001
	No	339 (52.2)	206 (63.4)	133 (40.9)	
Do you think that you have enough information about the COVID‐19 vaccine?	Yes	143 (22.0)	33 (10.2)	110 (33.80	< 0.001
	No	507 (78.0)	292 (89.8)	215 (66.2)	
Do you think that the COVİD‐19 vaccine carries the possibility of Harm to your baby?	Yes	473 (72.8)	240 (73.8)	233 (71.7)	0.537
	No	177 (27.2)	85 (26.2)	92 (28.3)	
If the COVID‐19 vaccine were recommended for pregnant women, would you have been vaccinated?	Yes	158 (24.4)	49 (15)	102 (31.5)	0.001
	No	342 (52.6)	228 (70.1)	114 (35)	
	I have been vaccinated	150 (23)	48 (14.9)	109 (33.5)	

^a^ Chi‐squared test.

In total, 58.6% of pregnant women had some level of awareness about the COVID‐19 vaccine. Of these, 48.9% of pregnant women in Zahedan did not know the vaccine's effectiveness, while 58.2% of those in Tabriz had some awareness of its impact. Additionally, 54.2% of pregnant women believed that the vaccine could affect the fetus. Independent *t*‐test results revealed a significant difference in the mean awareness scores between the two groups of women (*p* < 0.05). The mean awareness score for pregnant women in Zahedan was 7.83 (SD = 2.01), while in Tabriz, it was 9.08 (SD = 2.19), indicating that the average awareness score was lower among pregnant women in Zahedan (Table 3).

**Table 3:** Distribution of each awareness item and differences in the two cities.

Questions	Answer	Total	Zahedan	Tabriz	*p* value^a^
Do you know about the COVID‐19 vaccine?	Yes	95 (14.6)	46 (14.6)	49 (15.1)	< 0.001^a^
	No	174 (26.8)	115 (35.4)	59 (18.2)	
	Somewhat	381 (58.6)	164 (50.5)	217 (66.8)	
Do you know about the effectiveness of COVID‐19 vaccine?	Yes	83 (12.8)	33 (10.2)	50 (15.4)	< 0.001^a^
	No	245 (37.7)	159 (48.9)	86 (26.5)	
	Somewhat	322 (49.5)	133 (40.9)	189 (58.2)	
Is it dangerous to use overdose vaccines?	Yes	208 (32.0)	95 (29.2)	113 (34.8)	0.240^a^
	No	75 (11.5)	42 (12.9)	33 (10.2)	
	Don't know	367 (56.5)	188 (57.8)	179 (55.1)	
Does vaccination increase allergic reactions?	Yes	105 (16.2)	37 (11.4)	68 (20.9)	0.004^a^
	No	84 (12.9)	44 (13.5)	40 (12.3)	
	Don't know	461 (70.9)	244 (75.1)	217 (66.8)	
Does vaccination increase autoimmune (unknown) diseases?	Yes	188 (28.9)	63 (19.4)	125 (38.5)	< 0.001^a^
	No	71 (10.9)	39 (12.9)	32 (9.8)	
	Don't know	391 (60.2)	223 (68.6)	168 (51.7)	
Does the COVID vaccination during pregnancy affect the fetus?	Yes	352 (54.2)	171 (52.6)	181 (55.7)	0.618^a^
	No	43 (6.6)	24 (7.4)	19 (5.8)	
	Don't know	255 (39.2)	130 (40.0)	125 (38.5)	
Does the regular flu vaccine have a protective role against COVID?	Yes	88 (13.5)	49 (15.1)	39 (12.0)	0.009^a^
	No	187 (28.8)	76 (23.4)	111 (34.2)	
	Don't know	375 (57.7)	200 (61.5)	175 (53.8)	
Is there an effective way to slow down the spread of COVID?	Yes	302 (46.5)	124 (38.2)	178 (54.8)	< 0.001^a^
	No	267 (41.1)	174 (53.5)	93 (28.6)	
	Don't know	81 (12.5)	27 (8.3)	54 (16.6)	
Total scores of awareness, mean (SD)		8.45 (1.20)	7.83 (101)	9.08 (1.19)	< 0.001^b^

^a^ Chi‐squared test.

^b^ Independent *t*‐test.

Table 4 reveals that, except for perceived barriers, the average scores for the other components of health belief were higher among pregnant women in Tabriz compared to those in Zahedan, and independent *t*‐tests demonstrated significant differences in the mean scores for all components between pregnant women in these two cities (*p* < 0.05).

**Table 4:** Distribution and comparison of the dimensions of health beliefs in two cities Zahedan and Tabriz.

Dimensions of Health Belief Model	Questions	Answer	Total	Zahedan	Tabriz	*p* value^a^
Susceptibility	Are you concerned about getting COVID‐19	Not concerned	200 (30.7)	125 (38.4)	75 (23.1)	< 0.001
		Concerned	450 (69.3)	200 (61.6)	250 (76.9)	
	Are you concerned about unborn baby getting COVID‐19	Not concerned	154 (23.6)	98 (30.1)	56 (17.2)	
		Concerned	496 (76.4)	227 (69.9)	269 (82.8)	
	Susceptibility, mean (SD)			1.20 (0.42)	1.90 (0.31)	
Severity	If a pregnant woman gets COVID‐19, she is more likely to have severe illness	Disagree	215 (33.1)	118 (36.3)	97 (29.8)	0.04
		Agree	435 (66.9)	207 (63.7)	228 (70.2)	
	If a pregnant woman gets COVID‐19, the illness could harm her unborn baby	Disagree	184 (28.3)	105 (32.3)	79 (28.2)	
		Agree	466 (71.7)	220 (67.7)	246 (75.7)	
	Severity, mean (SD)			1.10 (0.44)	1.80 (0.42)	
Barriers	Vaccine can cause a person to get sick with COVID‐19	Disagree	208 (32.0)	89 (27.4)	119 (36.6)	0.04
		Agree	442 (68.0)	236 (72.6)	206 (63.4)	
	Vaccine is not safe during pregnancy	Disagree	188 (28.9)	76 (23.4)	112 (34.5)	
		Agree	462 (71.1)	249 (76.6)	213 (65.5)	
	Vaccine is not an effective way to prevent a pregnant woman from getting COVID‐19	Disagree	179 (27.5)	65 (20.0)	114 (35.1)	
		Agree	471 (72.5)	260 (80.0)	211 (64.9)	
	Barriers, mean (SD)			2.60 (0.57)	1.60 (0.89)	
Benefits	Giving vaccine to a pregnant woman will benefit her fetus and baby	Disagree	475 (73.1)	257 (79.1)	218 (67.1)	< 0.001
		Agree	175 (26.9)	68 (20.9)	107 (32.9)	
	Getting vaccine during pregnancy is a benefit for the pregnant woman	Disagree	378 (53.6)	223 (68.6)	155 (47.6)	
		Agree	302 (46.4)	102 (31.4)	200 (52.4)	
	Vaccine could protect the baby during the first months of life	Disagree	230 (35.4)	132 (40.6)	98 (30.2)	
		Agree	420 (64.6)	193 (59.4)	227 (68.9)	
	Benefits, mean (SD)			0.40 (0.51)	1.60 (0.84)	
Cues to action	If doctors recommended vaccine, I would get vaccinated	Disagree	188 (28.9)	108 (33.2)	80 (24.7)	0.048
		Agree	462 (71.1)	217 (66.8)	245 (75.3)	
	If a nurse or midwife recommended vaccine, I would get vaccinated	Disagree	230 (35.4)	122 (37.5)	108 (33.2)	
		Agree	420 (64.6)	203 (62.5)	217 (66.8)	
	If family members recommended vaccine, I would get vaccinated	Disagree	155 (23.8)	54 (16.6)	101 (31.1)	
		Agree	495 (76.2)	271 (83.4)	224 (68.9)	
	If the Media recommended vaccine, I would get vaccinated	Disagree	272 (41.8)	149 (45.8)	123 (37.8)	
		Agree	378 (58.2)	176 (54.2)	202 (62.2)	
	Cues to action, mean (SD)			2.60 (0.51)	3.80 (0.42)	

^a^ Independent *t*‐test.

Other findings from this table indicate that 76.4% of the total pregnant mothers are concerned about the possibility of their fetus contracting COVID‐19. 66.9% believe that if a pregnant woman contracts COVID‐19, the severity of the illness is greater. Additionally, 76.6% and 65.5% of participants from Zahedan and Tabriz, respectively, are unaware of the safety of COVID vaccination during pregnancy. Furthermore, 83.4% of pregnant women in Zahedan are likely to get vaccinated if recommended by family members, while in Tabriz, 75.3% consider a doctor's recommendation to be influential (Table 4).

A multinomial logistic regression model showed that the likelihood of vaccine acceptance increases with the mother's employment status (OR = 11.53, 95% CI: 4.81–27.63), higher maternal education (OR = 6.97, 95% CI: 3.11–15.63), higher income (OR = 1.60, 95% CI: 1.07–2.55), and an increase in awareness level (OR = 1.37, 95% CI: 1.03–1.52). Additionally, the likelihood of vaccine acceptance increases with an increase in perceived sensitivity (OR = 2.55, 95% CI: 0.47–3.76), perceived benefits (OR = 2.22, 95% CI: 1.06–4.61), and cues to action (OR = 2.01, 95% CI: 1.03–3.92), and decreases with perceived barriers (OR = 0.42, 95% CI: 0.18–1.12) (Table 5).

**Table 5:** Predictors for acceptance of vaccine based on multivariable logistic regression with adjustment of sociodemographic and obstetric characteristics, awareness and five dimensions of Health Beliefs Model.

Characteristic	Acceptance of vaccine	
	Adjusted	*p* value^a^
	OR (95% CI)	
Age category (reference: < 20)	—	—
20–29	1.437 (0.78–2.639)	0.243
30–39		0.332
≥ 40		0.147
Mother's employment status (reference: housewife)	—	—
Employed	11.538 (4.81–27.637)	< 0.001
Mother's educational status (reference: primary school)	—	—
Secondary school	0.829 (0.38–1.767)	0.627
Diploma	3.024 (1.58–5.753)	0.001
University	6.977 (3.11–15.632)	< 0.001
Economic status (reference: less than enough)	—	—
More than enough	12.779 (1.16–140.305)	0.037
Enough	1.660 (1.07–2.556)	0.021
Spouse's educational status (reference: primary school)	—	—
Secondary school	0.820 (0.46–1.448)	0.494
Diploma	0.210 (0.658–0.379)	0.210
University	0.483 (0.77–0.376)	0.483
Spouse's employment status (reference: jobless)	—	—
Employed	1.848 (0.69–4.894)	0.216
Self‐employed	2.427 (0.78–7.510)	0.124
Gravida (reference: > 1)	—	—
2	1.342 (0.79–2.27)	0.273
> 3	1.059 (0.63–1.77)	0.829
Gestational age (reference: ≥ 14)	—	—
15–28	0.935 (0.32–2.70)	0.901
≥ 29	1.486 (0.03–4.38)	0.473
Awareness	1.370 (1.23–1.52)	< 0.001
Health beliefs		
Susceptibility	2.55 (0.47–3.76)	0.02
Severity	1.02 (0.24–2.81)	0.765
Barriers	0.42 (0.18–1.12)	0.043
Benefits	2.22 (1.06–4.61)	0.033
Cues to action	2.01 (1.03–3.92)	0.041

^a^ Multivariable logistic regression.

## 4 Discussion

According to the authors' examination, the present study is the first of its kind to investigate the factors related to the acceptance of the COVID‐19 vaccine among pregnant women in both high and low‐fertility regions in Iran. In this multicenter cross‐sectional study conducted in healthcare centers across two major provinces in Iran, 52.6% of pregnant women reported vaccine nonacceptance during pregnancy. The highest rate of vaccine nonacceptance was observed in Zahedan.

In a survey conducted across 16 countries, the acceptance rate of vaccines among pregnant women was reported to be 52% [12]. Similarly, Moini et al. [25] reported a 50% acceptance rate of vaccines among pregnant women residing in Tehran. It is important to note that pregnant women are considered a priority population for seasonal influenza vaccination by the World Health Organization due to their increased susceptibility to infection [24]. Nevertheless, the critical point to consider is that a significant percentage of pregnant women in Zahedan who participated in our study were unwilling to receive the influenza vaccine, despite recommendations. This contrasts with the situation in Tabriz, where more than half of pregnant women had already received the influenza vaccine.

It appears that doubt and hesitation regarding the effectiveness of any vaccine can play a role in its utilization. The results of our study indicated that the level of education has a significant impact on vaccine acceptance. In this research, pregnant women in Tabriz had higher educational levels compared to pregnant women in Zahedan. Similar findings have been reported by Jones and Fridman in their studies, where higher education was associated with better vaccine acceptance [26, 27].

It has been suggested that lower education levels may lead to vaccine hesitancy due to gaps in knowledge regarding the effectiveness and safety of vaccines or inflexible attitudes toward vaccination [28]. However, Tao et al. [24] reported a lower vaccine acceptance rate among educated pregnant women, as assessed using the Health Belief Model. Wang et al. [29] also reported a lower vaccine acceptance rate among nurses due to doubts about effectiveness, safety, and efficacy. Researchers emphasize the importance of receiving accurate information, especially from credible sources, as receiving incorrect information can lead to increased anxiety and concern about vaccine side effects, even among individuals with higher education levels, particularly during the initial stages of vaccine development.

The present study found that higher awareness scores are linked to better vaccine acceptance, consistent with some studies [24, 30]. However, it contradicts a study by Dhakal et al., which observed an inverse relationship between good awareness and COVID‐19 vaccine acceptance. They suggested that pregnant women with more knowledge about COVID‐19 might have greater access to various information sources, potentially leading to incomplete and inaccurate information and misconceptions about vaccine effectiveness and safety [31].

Our study results indicate that in Tabriz, pregnant mothers exhibit higher sensitivity to the disease and higher perceived benefits of vaccination. Conversely, in pregnant mothers from Zahedan, perceived barriers to vaccine acceptance were more pronounced, and trust in family members for vaccination decisions was more prominent. According to the results of our study, individuals with higher perceived susceptibility to infection or a positive attitude toward vaccination benefits were more likely to accept the vaccine. Conversely, higher perceived barriers led to a negative attitude toward vaccination. This finding is in line with other studies [24, 32], although there were studies that did not find a significant association between perceived susceptibility and vaccine acceptance [33].

The importance of individuals having a good understanding of vaccine effects and their impact on vaccine acceptance is supported by various studies [30, 31]. In this study, 76.6% of the mothers in Zahedan believed that the vaccine was not safe. Considering the natural fears of mothers, reliable sources of information must be made available. Therefore, counseling and recommendations provided by healthcare providers during prenatal visits are crucial.

However, the results indicate that these individuals rely more on their families rather than trusting the healthcare providers. In a study conducted in this province, the rate of prenatal care was reported to be 44% or less, which is lower than other regions in the country [34]. Since social and economic factors are known to influence vaccine acceptance [35, 36], and Zahedan is a deprived provincial center in Iran, it seems that the lack of access to quality healthcare and obtaining information from individuals with potentially lower health literacy may lead to a reduced willingness to accept the vaccine in this population compared to other regions of Iran.

Therefore, healthcare professionals and policymakers should engage in more comprehensive planning for the development of health messages and educational campaigns emphasizing the benefits of vaccination, particularly targeting vulnerable populations, such as pregnant women. Additionally, in areas with lower educational levels or lower health literacy, efforts should be made to increase health knowledge through culturally sensitive and emotionally resonant visual and language communication strategies.

Furthermore, in this region, there is a traditional and religious context in which women's decision‐making is often dependent on men. This can lead to misconceptions and their impact on vaccine hesitancy. Studies have shown that individuals with lower levels of education or literacy tend to be more strongly tied to Islamic beliefs and may perceive vaccines as a Western conspiracy aimed at harming them and diverting them from their religious path [37, 38]. Therefore, designing health‐centered interventions for pregnant women to correct misconceptions and empower them in decision‐making for the well‐being of themselves and their families is of paramount importance.

One of the strengths of the present study is that it was conducted for the first time in two cities in Iran with different fertility rates, Zahedan with a higher fertility rate and Tabriz with a lower fertility rate. These two cities are in different regions of Iran and exhibit significant differences in terms of economic, social, and cultural aspects. However, the limitation of the study was that the samples were selected from healthcare centers, and other healthcare facilities, such as hospitals and physicians' offices, were not included in the assessment. Therefore, the results cannot be generalized to all pregnant women.

## 5 Conclusion

Based on the obtained results, most pregnant women believe that the COVID‐19 vaccine is not safe during pregnancy and cannot prevent the disease. However, factors such as the educational level of mothers, awareness, perceived disease sensitivity, and perceived vaccine benefits can influence their willingness to accept the vaccine. Additionally, the media, family members, and healthcare providers also play roles in vaccine acceptance. Therefore, a tailored and strategic approach is needed. It is recommended that policymakers and planners design and implement necessary interventions considering the conditions and needs of each region. These interventions may include raising awareness, providing scientific justifications, facilitating access to vaccines at various healthcare centers, and efforts to increase COVID‐19 vaccine acceptance. This is essential to prevent the consequences and mortality resulting from the disease, especially among pregnant women, who are considered a vulnerable population.

## Author Contributions

**Azita Fathnezhad‐Kazemi:** methodology, writing–review and editing, writing–original draft. **Lila Hadipour:** formal analysis, writing–review and editing. **Raziyeh Pirami:** formal analysis, writing–review and editing. **Somayyeh Khazaeian:** writing–original draft, writing–review and editing, supervision, methodology.

## Conflicts of Interest

The authors declare no conflicts of interest.

## Data Availability

The data are available to reproduce results on request from the corresponding author.
